# Nation‐wide cross‐sectional study of participation and predictors of enjoyment among Australian adult walking sport participants

**DOI:** 10.1002/ejsc.12246

**Published:** 2025-01-09

**Authors:** Jasmine M Petersen, Cath J Connolly, Lucy K Lewis

**Affiliations:** ^1^ Flinders University College of Nursing and Health Sciences Caring Futures Institute Adelaide South Australia Australia

**Keywords:** adults, enjoyment, physical activity, sport, walking sports

## Abstract

This study examined participation and predictors of walking sports enjoyment among Australian adult walking sport participants. An online cross‐sectional survey assessed walking sport participation, enjoyment, and barriers and motives to participation. Physical activity behavior and motivations were also assessed. The sample comprised 294 walking sport participants (M_age_ = 62.9 ± 10.5 years). Participants engaged in a variety of walking sports (e.g., football, netball, and basketball) and largely did so one occasion per week (for ≤3 h). Our findings suggest that typically walking sport participants are female (60.1%), aged ≥60 years (54.7%), in married/de facto relationships (73.4%), reside in higher socioeconomic status areas, and are sufficiently active (i.e., ≥150 min of activity per week; 91.0%). The most endorsed barrier to walking sport participation was physical health (19.7%), while fun/enjoyment (90.5%) was the most endorsed motive. The regression analyses showed that gender (being female; *β* = 0.17), older age (≥60 years; *β* = −0.21), and intrinsic motivation for physical activity (*β* = 0.23) were significant predictors of walking sport enjoyment. Ongoing efforts to ensure that walking sports are a positive experience for all are necessary. The provision of walking sport offerings that integrate elements (e.g., social connectedness) known to be of value to specific subpopulations may be important to fostering enjoyment.

## Highlights

- Fun/enjoyment was the most endorsed motive to walking sport participation.
- Being female and intrinsic motivation were positive predictors of walking sport enjoyment.
- Older age was a negative predictor of enjoyment.

## 1 INTRODUCTION

Globally, 1.4 billion adults (28%) are insufficiently active (World Health Organization, 2022) with higher rates of inactivity reported in high‐income Western countries (Australian Institute of Health and Welfare, 2023). In Australia, for example, over a third (35%) of the adult population do not meet the physical activity guidelines (i.e., 150 min of moderate‐to‐vigorous intensity physical activity per week) (Australian Institute of Health and Welfare, 2023). It is well documented that physical activity is associated with many physical and mental health benefits, including reducing the risk of cardiovascular disease, obesity, Type 2 diabetes, breast and colon cancer, depression, and anxiety (Rhodes et al., 2017). As such, improving physical activity engagement, both in Australia and more broadly, is critical.

Increasing sport participation is recognized as important to curbing population physical inactivity (World Health Organization, 2019). Recent evidence indicates that fewer than half (23.6%) of Australian adults (≥25 years) participate in sport, and participation declines with age (e.g., 17% of adults ≥55 years engage in sport) (Australian Sports Commission, 2024). Sport confers a range of benefits beyond that of physical activity, including enhanced social outcomes, such as fostering social support, connectedness, and belonging (Eather et al., 2023). On the other hand, it is recognized that there are many benefits of adults in sport, including acting as role models and mentors for younger members along with their engagement in volunteering, which is important to increasing organization/club capacity (Jenkin et al., 2017). Ongoing efforts to improve adult sport participation are necessary, particularly given that a lack of age‐appropriate sport offerings is cited as a prominent barrier to engaging this subpopulation (Jenkin, van Uffelen, et al., 2021). The World Health Organization's Global Action Plan on Physical Activity 2018–2030, targeted at establishing more active societies, recommends the development of approaches aimed at increasing access to, and opportunities for, sport participation across all ages and abilities (World Health Organization, 2019).

Modified sports, such as walking sports, provide inclusive and accessible opportunities for sport participation across the population more broadly (e.g., inactive, older, or less mobile subpopulations). The growing popularity of walking sports has fueled the emergence of a small body of research in relation to such sports. There is some evidence in relation to the beneficial outcomes of walking sport participation (Corepal et al., 2020). For example, walking football is shown to be a moderate‐to‐vigorous intensity activity (Harper et al., 2019), associated with reductions in body fat mass (Arnold et al., 2015), along with improvements in physical fitness parameters (e.g., balance and leg strength) and mental health outcomes (e.g., depression) (Yüce & Saygın, 2024). Other research pertaining to walking sports has largely focused on the experiences (or perceptions) of those participating in these sports (Cholerton et al., 2019, 2021; Sivaramakrishnan, Phoenix, et al., 2023). For example, Cholerton et al. (2019) reported that adults' (55–75 years) initial experiences of participating in walking football in the UK were both positive (physical and mental health benefits and social connections) and negative (injury and inconvenient timing). More recently, Cholerton et al. (2021) identified several factors that may support continued participation in walking sports, including individual influences (e.g., enjoyment), social influences (e.g., club culture), and session‐specific factors (accessible facilities). To date, however, there is little knowledge pertaining to those that choose to engage in walking sports, nor insight into factors shown to underlie positive experiences in such sports.

Physical activity is imperative to optimizing population physical and mental health outcomes (Rhodes et al., 2017). Walking sports are an important and novel approach to supporting physical activity engagement among subpopulations that are most inactive, such as middle‐aged and older adults. There is, however, a paucity of research pertaining to walking sport participation, and more specifically, such participation in the Australian context; wherein, the popularity of walking sports has grown immensely. Therefore, this study aimed to examine walking sport participation among Australian adults engaging in such sports. It further aimed to explore potential predictors of walking sports enjoyment, a construct shown to be critical to facilitating walking sport participation (Cholerton et al., 2021) and participation in sports more broadly (Crossman et al., 2024). This study has important implications for strengthening walking sport offerings and may be an important catalyst to improving population physical activity engagement.

## 2 METHOD

### 2.1 Design

This study used an observational cross‐sectional design. Ethical approval was gained from the Flinders University Humans Research Ethics Committee (protocol no. 8542), and all participants provided informed consent electronically. The reporting of this study complies with STROBE guidelines (Vandenbroucke et al., 2007) (Supplementary file 1).

### 2.2 Participants

Eligible participants were those currently participating in walking sports in Australia or had done so recently (during the prior 12 months). Walking sport organizations (*n* = 170) were contacted via email and invited to disseminate the study information (and survey link) to their member database. Organizations that did not respond were followed up on one subsequent occasion. A total of 325 individuals commenced the survey of whom 31 did not provide data in relation to walking sport participation. This resulted in a final sample of 294 walking sport participants.

### 2.3 Procedure

Data were collected between February 2022 and January 2024 using an online survey administered via the Qualtrics platform. The survey (approx. 10 min) incorporated measures pertaining to sociodemographic and health information, physical activity behavior (and motivations), and walking sport participation.

### 2.4 Measures

#### 2.4.1 Sociodemographics

Participants were asked to report their age, gender, residential postcode, marital status, highest education level, and employment status. Socioeconomic status was determined from a residential postcode using the Australian Bureau of Statistics Socioeconomic Indexes for Areas (SEIFA) Index of Relative SocioEconomic Advantage and Disadvantage (ABS, 2018). Higher values represent a higher socioeconomic status decile (range 1–10) (Australian Bureau of Statistics, 2018).

#### 2.4.2 Health information

The Functional Comorbidity Index (Groll et al., 2005) was used to determine the number of comorbidities. Body mass index (BMI) was calculated by dividing participants' self‐reported weight (kg) by height in meters squared (kg/m^2^) (Department of Health and Aged Care, 2021). Participants were asked to rate their overall health with response options ranging from excellent to poor. Response options were categorized as excellent/very good, good, and fair/poor (Gandhi et al., 2020).

#### 2.4.3 Physical activity behavior

Self‐reported physical activity was assessed using the Active Australia Survey (AIHW, 2003). Participants were asked to specify the frequency and total duration (minutes) of participation in the following types of activity (in the previous week): (1) walking continuously for at least 10 min, (2) vigorous gardening or heavy work around the yard, (3) vigorous physical activity, and (4) other moderate physical activities (AIHW, 2003). Weekly moderate‐to‐vigorous physical activity (MVPA) was calculated by summing minutes spent walking in moderate activity and in vigorous activity (AIHW, 2003).

#### 2.4.4 Physical activity motivations

The Behavioral Regulation Exercise Questionnaire (BREQ‐2) was used to assess motivation for physical activity based on Self‐Determination Theory (Markland & Tobin, 2004). The questionnaire incorporates 19 items, comprising five subscales assessing intrinsic motivation (4 items), identified regulation (4 items), introjected regulation (3 items), external regulation (4 items), and amotivation (4 items). Participants rated items on a five‐point Likert scale from 0 (not true) to 4 (very true), and a score for each subscale was generated by averaging the representative items. In the current study, the reliability of the subscales ranged from *α* = 0.67 for amotivation to *α* = 0.88 for intrinsic motivation.

#### 2.4.5 Walking sport participation

Participants self‐reported their walking sport participation. They were asked to indicate their main walking sporting code, frequency, (response options ranging from 1 day to 7 days) and duration of participation per week (response options ranging from 1 h to >5 h). Participants also indicated their motives (e.g., physical health benefits, fun/enjoyment, and competition) and barriers (e.g., lack of time, competing demands, and cost) to participating in walking sports from a list of response options including the option to specify “Other”. The response options were derived from existing research pertaining to walking sport participation (Cholerton et al., 2019; Corepal et al., 2020; Jenkin, Hilland, & Eime, 2018; Kinnafick et al., 2021). A further item was used to assess participants' intentions to continue participating in walking sports in the subsequent 12 months (response options; yes/no).

#### 2.4.6 Walking sport enjoyment

The Physical Activity Enjoyment Scale was used to assess participants' walking sport enjoyment (Mullen et al., 2011). The scale incorporates eight items that are rated on a seven‐point Likert scale, for example, from one *(it is no fun at all*) to seven (*it is a lot of fun*). The items were averaged with higher scores indicating higher enjoyment of walking sports. Internal reliability in this study was *α* = 0.76.

### 2.5 Data analysis

Statistical analyses were conducted using SPSS statistical software version 29. Descriptive statistics were generated for all variables. A multiple linear regression was conducted to determine the predictors of walking sport enjoyment. The regression model incorporated physical activity motivations, sociodemographic characteristics (e.g., age and gender), and health information (comorbidities, BMI, and perceived health). Assumptions for linear regression were not violated (i.e., independence of observations, linearity and homoscedasticity, normality, multicollinearity, and undue influence). A power analysis (G*Power) for linear regression indicated that the sample size (*n* = 294) was adequate to detect a medium‐sized effect (*f* ^2^ = 0.15) with 80% power and the alpha level of 0.05.

## 3 RESULTS

### 3.1 Descriptive characteristics

The sample comprised 294 Australian adults (60.0% female), with an average age of 62.9 years (SD 10.5; range 25–83 years) (see Table 1). Participants were predominately married/in de facto relationships (73.1%). Fewer than half had a university education (41.5%) or were (currently) in paid employment (41.8%). Mean BMI was 27.6 kg/m^2^ (*SD* = 5.2; range 17.0–47.6 kg/m^2^). On average, participants self‐reported at least one comorbidity (*M* = 1.7, *SD* = 1.5) and most perceived their general health as good/excellent (71.1%). Participants resided in all Australian states and territories (except Tasmania) and were predominately in higher socioeconomic status areas (SEIFA ≥ 5; 85.0%).

**TABLE 1:** Descriptive characteristics.

Characteristic	Overall (*n* = 294)
Age (years), *n* (%)^a^	
< 60 years	73 (25.0)
≥ 60 years	161 (54.7)
Gender, *n* (%)	
Female	176 (60.1)
Male	117 (39.9)
Perceived general health, *n* (%)	
Poor/fair	12 (4.1)
Good	72 (24.5)
Very good/excellent	209 (71.1)
No. comorbidities, *M* (SD)	1.7 (1.5)
BMI, *M* (SD)	27.6 (5.2)
SEIFA index of relative advantage and disadvantage decile, *M* (SD)	7.4 (2.4)
Relationship status, *n* (%)	
Single	68 (23.1)
Married/de facto relationship	215 (73.4)
Highest education, *n* (%)	
Year 12 (or lower)	75 (25.5)
Postsecondary diploma or certificate	97 (33.0)
Bachelor or postgraduate degree	122 (41.5)

^a^ Total number in each row may not equate to 294 due to missing responses.

### 3.2 Engagement in physical activity

On average, participants self‐reported engaging in 8.8 h per week (528 ± 393 min/week) of MVPA and 91.0% were categorized as sufficiently active (≥150 min of activity per week) (see Table 2). The sample reported higher levels of autonomous (rather than controlled) forms of motivation for physical activity.

**TABLE 2:** Descriptive data in relation to physical activity and walking sport engagement.

Characteristic	Overall (*n* = 294)
MVPA (min/week), *M* (SD)	528 (393)
Sufficient activity, *n* (%)	
Sedentary	2 (0.7)
Insufficiently active	23 (8.3)
Sufficiently active	253 (91.0)
Physical activity motivation	
Intrinsic motivation	3.3 (0.7)
Identified regulation	3.2 (0.7)
Introjected regulation	1.2 (0.9)
External motivation	0.3 (0.5)
Amotivation	0.1 (0.3)
Walking sport participation, *n* (%)	
Days/week	
1–3 days/week	291 (99.0)
> 3 days/week	3 (1.0)
Hours/week	
1–3 h/week	274 (93.2)
> 3 h/week	20 (6.8)
Walking sport enjoyment	5.6 (1.0)

### 3.3 Walking sport participation

Participants largely reported engaging in walking sports one (72.1%) or 2 days (23.5%) per week and did so for ≤3 h per week (93.2%) (see Table 2). Sporting codes represented in the sample included football (soccer; 43.5%), netball (24.8%), basketball (18.4%), hockey (11.9%), and tennis (0.3%). The most endorsed motives for participation in walking sports were (1) fun/enjoyment (90.5%), (2) physical health benefits (83.0%), (3) social benefits (79.3%), and (4) mental health benefits (54.1%). The highest perceived barriers to walking sport participation were physical health (19.7%), followed by bad weather (15.6%), competing demands (14.6%), concern about injury (13.9%), and lack of time (11.9%). Participants indicated high perceived enjoyment of walking sports (PACES; *M* = 5.6 ± 1.0; range 1–7, and most (89.1%) reported intentions to continue participating.

### 3.4 Predictors of walking sport enjoyment

Results of the multiple regression analysis are presented in Table 3. The regression model accounted for 14.6% of the variance in walking sport enjoyment (*R* ^2^ = 0.146) and was significant, *F*(16, 211) = 2.2, *p* = 0.005. The significant positive predictors of walking sport enjoyment were gender (being female) and intrinsic motivation for physical activity. Older age (≥60 years) was a significant negative predictor of walking sport enjoyment.

**TABLE 3:** Linear regression examining predictors of walking sport enjoyment.

	*β*	*p*‐value	95% CI
Age (<60 years if ref)			
≥ 60 years	−0.21	**0.016**	[−0.38, −0.04]
Gender			
Female (vs. Male)	0.17	**0.011**	[0.04, 0.30]
Perceived health (very good/excellent is ref)			
Good	−0.07	0.301	[−0.22, 0.07]
Poor/fair	−0.12	0.087	[−0.25, 0.02]
No. of comorbidities	0.02	0.837	[−0.13, 0.17]
BMI (kg/m^2^)	0.07	0.389	[−0.08, 0.21]
SEIFA rank of location	−0.03	0.701	[−0.17, 0.11]
Relationship status			
Married/de facto relationship (vs. single)	−0.01	0.854	[−0.14, 0.12]
Education (Year 12 or lower is ref)			
Postsecondary diploma or certificate	−0.02	0.839	[−0.18, 0.15]
Bachelor or postgraduate degree	−0.14	0.107	[−0.31, 0.03]
Currently in paid employment (yes is ref)	0.13	0.123	[−0.03, 0.29]
Physical activity motivations			
Intrinsic motivation	0.23	**0.009**	[0.06, 0.41]
Identified regulation	0.005	0.956	[−0.18, 0.19]
Introjected regulation	0.05	0.541	[−0.11, 0.20]
External regulation	−0.009	0.905	[−0.16, 0.14]
Amotivation	0.04	0.550	[−0.10, 0.19]

*Note*: Bold denotes statistical significance p < 0.05.

## 4 DISCUSSION

This study provides the first nation‐wide examination of walking sport participation among Australian adults. We also offer insights into predictors of walking sport enjoyment, a construct known to be critical to facilitating sport participation (Crossman et al., 2024). This study has important implications for improving sport participation across the lifespan.

This study suggests that Australian adults engage in a variety of walking sports (e.g., football, netball, and basketball) on typically one occasion per week (for ≤3 h). Our findings also offer novel insights into the sociodemographic profile of those that engage in walking sports. Participants were predominately female, aged ≥60 years, married/in de facto relationships, and resided in higher socioeconomic status areas. Interestingly, one‐quarter of walking sport participants were aged <60 years. Existing research pertaining to walking sports has largely focused on older adults (e.g., adults 55–75 years; Cholerton et al., 2021). The present study indicates that walking sports may also be an appropriate (and important) strategy to engage young and middle‐aged adults in sport, especially given the prevalence of physical inactivity among these subpopulations (Australian Institute of Health and Welfare, 2023). It has however been suggested that the delivery of modified sports (e.g., walking sports) is limited, and instead there has been a continued focus on providing opportunities to engage children and young people in sport (and this is reflected in sport policy) (Jenkin, van Uffelen, et al., 2021). Given that the population is aging rapidly and physical activity and sport participation are critical to healthy aging (Abud et al., 2022), we must ensure that appropriate, age‐friendly playing opportunities are offered. Sporting organizations and clubs are known to contend with many barriers (e.g., competing priorities and limited resources) (Petersen et al., 2024) that may undermine their capacity to provide sport offerings that cater to all subpopulations. As such, it is acknowledged that systemic change, including reform to sport policy for instance, may be key to prioritizing the provision of appropriate opportunities to support adult sport participation (Jenkin, van Uffelen, et al., 2021).

On average, participants reported engaging in 528 min of MVPA per week and over 90% were categorized as sufficiently active (i.e., meeting physical activity guidelines of ≥150 min of activity per week). Therefore, they were markedly more active than the general Australian population (Australian Institute of Health and Welfare, 2023). They also reported higher MVPA relative to other research (using the Active Australia Survey) in adult populations broadly (357–395 min MVPA/week; Quinlan et al., 2021; Zafiropoulos et al., 2019). This could, however, be attributed to our sample comprising only those participating in walking sports, given that walking sports are a moderate‐to‐vigorous intensity activity (Harper et al., 2019) and sport participation (broadly) is known to contribute to the accumulation of MVPA (Ridley et al., 2018; Toivo et al., 2023). Future research could usefully provide greater insight into the intensity of physical activity behaviors during walking sport participation (e.g., accelerometer‐derived time spent in MVPA). Nevertheless, it is well‐established that physical activity (including walking sports) confers many beneficial health outcomes (Rhodes et al., 2017), and as such, it is perhaps not surprising that most participants positively perceived their general health and had few comorbidities. This adds to the ever‐growing body of evidence pertaining to the value of physical activity engagement (Rhodes et al., 2017) and underscores the importance of accessible and inclusive opportunities (e.g., walking sports) to facilitate active lifestyles. As in the present study, existing research has largely focused on those already participating in walking sports (Cholerton et al., 2021; Mulvenna & Leslie‐Walker, 2021; Sivaramakrishnan, Phoenix, et al., 2023). We must now ascertain the potential (e.g., acceptability, feasibility, and sustainability) of walking sports to support physical activity engagement among inactive populations. Older adults in residential aged care settings, for example, are shown to spend much of their waking time sedentary (9.6 h/day; 85.0%) (Parry et al., 2018) and have high levels of physical frailty (Milte et al., 2022). The adaption of walking sport offerings for provision in such settings, that are anticipated to rapidly increase in demand (United Nations Department of Economic and Social Affairs, 2019), may be key to optimizing the physical functioning and quality of life of the aging population.

This study also sheds light on barriers and motives to walking sport participation. Our findings indicate that physical health, bad weather, and competing demands are prominent barriers to walking sport participation among Australian adults. Other research has similarly documented that physical health plays an important underlying role in walking sport participation (Jenkin, Hilland, & Eime, 2018; Sivaramakrishnan, Quested, et al., 2023). Sivaramakrishnan, Quested, et al. (2023), for example, reported that perceived health status was a key predictor of intentions to participate in walking sports, and more specifically, those with poorer perceived health were less inclined to participate. As such, individual influences (i.e., physical health) along with program‐related factors (e.g., scheduling, appropriate facilities for inclement weather) are important considerations in the development and implementation of walking sport programs in the future.

Our findings further indicate that physical health and social benefits are key motives for walking sport participation. Existing research also suggests that intrapersonal (e.g., improved physical and mental health outcomes) and interpersonal factors (e.g., social opportunities) are also important to reengaging older adults in community sport (Jenkin, Eime, et al., 2021). Given a lack of appropriate (or age‐specific) playing opportunities is a key reason for sport drop‐out among older adults (Jenkin, Eime, et al., 2021), walking sports may provide an important strategy to support older adults sustained engagement (e.g., ≥3 years/seasons of continuous participation in the same sport) or re‐engagement in sports (e.g., reenrolment in organized sports following ≥12 months absence) (Kay et al., 2024). Notably, almost all participants reported fun and enjoyment as motivators for their participation. This fits with existing research in relation to motives for both walking sport (Cholerton et al., 2021; Jenkin, Hilland, & Eime, 2018) and general sport participation across the lifespan (Crossman et al., 2024; Eime et al., 2024). For instance, Cholerton et al. (2021) cited enjoyment as critical to the sustained participation (≥6 months) of older British adults in walking football; this is particularly important given that sustained physical activity is associated with superior health outcomes (Rhodes et al., 2017). Insight into factors that foster walking sport enjoyment may therefore be key to supporting walking sport initiation and sustained participation.

The present study offers novel insights into factors that are linked to walking sport enjoyment. Our findings indicate that older age predicts lower walking sport enjoyment. Strategies to enhance older adults' experiences in walking sports are critical, particularly given they are a highly inactive (and sedentary) subpopulation (Dohrn et al., 2020). Intergenerational physical activity programs, for example, have been shown to be appealing to, and valued by, older adults (Buonsenso et al., 2021; Jenkin, Eime, et al., 2018). They are also found to be acceptable to younger generations (Schroeder et al., 2017), and notably, there is evidence to suggest they may foster enjoyment of, and sustained participation in, organized sports among younger people (Morgan et al., 2024). Walking sport offerings could therefore be enhanced by integrating intergenerational opportunities for engagement, and this will be beneficial to attracting and retaining both younger and older generations.

We further found that gender, and more specifically being female, predicts higher perceived enjoyment of walking sports. This is a novel finding given that it is well documented that fewer females participate in organized sports across the lifespan (relative to their male counterparts) (Australian Sports Commission, 2024) and are also more likely to drop out of such sports (Eime et al., 2020). The present study suggests that walking sports may be an appealing and appropriate approach to supporting female adult engagement (or re‐engagement) in sports. This could in part be underpinned by our finding that intrinsic motives for physical activity engagement positively predicted walking sport enjoyment. Current walking sport offerings are, for example, cited to foster a range of social outcomes (e.g., connectedness and belonging) (Mulvenna & Leslie‐Walker, 2021) that are known to be of value to, and motivate sport participation, among females (to a greater extent than males) (Crossman et al., 2024; Stenner et al., 2020). On the other hand, males are shown to be motivated by the competitive aspects of a sport offering (Ley, 2020), which are said to be lacking in current walking sport programs (Corepal et al., 2020). Our findings speak to the importance of walking sports offerings integrating elements (e.g., social and competitive) that are known to be of value to specific subpopulations to facilitate positive experiences and ultimately sustained participation.

### 4.1 Implications

The present study has important design implications for strengthening walking sport offerings. More specifically, sporting organizations (or clubs) should ensure that walking sport provisions are appropriate for, and inclusive of, all adult populations (e.g., scheduling). The adaption of walking sport offerings for specific settings (e.g., aged care) or subpopulations (e.g., sedentary or inactive) warrants consideration in future. Our findings highlight the importance of ensuring that walking sports are a positive experience (e.g., fun/enjoyable) for all. They further suggest that one‐size may not fit all when seeking to foster such enjoyment. For instance, integrating social elements (e.g., “come and try sessions” and provision of food and beverages) known to align with the key motives for sport participation among adult females (Crossman et al., 2024; Stenner et al., 2020) may be important to initiating (and sustaining) walking sport engagement among this demographic. While among older adults, the integration of intergenerational opportunities for participation may, for example, be necessary to improve their experiences in walking sports. Finally, this study suggests that walking sports may be appropriate (and accepted) by the population broadly (i.e., diversity in sample sociodemographics). Ongoing efforts to improve (and increase) such sport offerings may have immense public health impact and be key to establishing more age‐friendly sporting environments.

### 4.2 Strengths and limitations

This study has several strengths. It is the first to provide a nation‐wide examination of walking sport participation among Australian adults. Further strengths were the large sample size and the inclusion of reliable and validated survey instruments. Nevertheless, there are several limitations that must be acknowledged. We exclusively recruited current or recent walking sport participants, and this precluded comparisons with non‐walking sport participants. This study also incorporated a very physically active (and motivated) sample, and this may limit the generalizability of the findings.

## 5 CONCLUSIONS

The present study offers important insights into walking sport participation among Australian adults engaging in these sports. Our findings suggest that walking sports may be an appropriate (and acceptable) approach to improve physical activity engagement among the general adult population. Ongoing efforts to enhance enjoyment in walking sports are necessary. Integrating elements (e.g., social and competitive) known to be of value to specific subpopulations may be fundamental to fostering positive experiences in such sports. Future research should seek to further explore the potential of walking sport offerings to facilitate active lifestyles among inactive or sedentary subpopulations.

## CONFLICT OF INTEREST STATEMENT

The authors report there are no competing interests to declare.

## Supporting information

Supplementary Material

## Data Availability

The dataset used during the current study is available from the corresponding author on reasonable request.
