# Supplementary material for: Nation‐wide cross‐sectional study of participation and predictors of enjoyment among Australian adult walking sport participants
Source: Eur J Sport Sci. 2025 Jan 9;25(2):e12246. doi: 10.1002/ejsc.12246 (PMC11726637; doi:10.1002/ejsc.12246)
Supplement: Supplementary file 1 — Supplementary Material [file EJSC-25-e12246-s001.docx]

S1. STROBE Statement—checklist of items that should be included in reports of observational studies

|  | Item No. | Recommendation | Page  No. | Relevant text from manuscript |
| --- | --- | --- | --- | --- |
| **Title and abstract** | 1 | (*a*) Indicate the study’s design with a commonly used term in the title or the abstract | 1 | Title: A nation-wide cross-sectional study of walking sport: participation and predictors of enjoyment among Australian adults |
|  |  | (*b*) Provide in the abstract an informative and balanced summary of what was done and what was found | 2 | Clear Methods and Results reported in the Abstract |
| Introduction | | | |  |
| Background/rationale | 2 | Explain the scientific background and rationale for the investigation being reported | 3-4 | Clearly justified study aims in the context of the current evidence |
| Objectives | 3 | State specific objectives, including any prespecified hypotheses | 4 | ...this study aimed to examine walking sport participation among Australian adults, and explore potential predictors of walking sports enjoyment. |
| Methods | | | |  |
| Study design | 4 | Present key elements of study design early in the paper | 5 | This study used an observational cross-sectional design. |
| Setting | 5 | Describe the setting, locations, and relevant dates, including periods of recruitment, exposure, follow-up, and data collection | 5 | Data were collected between February 2022 and January 2024 using an online survey administered via the Qualtrics platform. |
| Participants | 6 | (*a*) *Cohort study*—Give the eligibility criteria, and the sources and methods of selection of participants. Describe methods of follow-up  *Case-control study*—Give the eligibility criteria, and the sources and methods of case ascertainment and control selection. Give the rationale for the choice of cases and controls  *Cross-sectional study*—Give the eligibility criteria, and the sources and methods of selection of participants | 5 | Eligible participants were those currently participating in walking sport in Australia (or had during the prior 12 months). |
|  |  | (*b*) *Cohort study*—For matched studies, give matching criteria and number of exposed and unexposed  *Case-control study*—For matched studies, give matching criteria and the number of controls per case |  |  |
| Variables | 7 | Clearly define all outcomes, exposures, predictors, potential confounders, and effect modifiers. Give diagnostic criteria, if applicable | 5-7 | Clearly defined outcomes. |
| Data sources/ measurement | 8* | For each variable of interest, give sources of data and details of methods of assessment (measurement). Describe comparability of assessment methods if there is more than one group | 5-7 | Clearly defined outcomes. |
| Bias | 9 | Describe any efforts to address potential sources of bias |  | N/A |
| Study size | 10 | Explain how the study size was arrived at | 7-8 | A power analysis (G*Power) for linear regression indicated that the sample size (*n* = 294) was adequate to detect a medium sized effect (*f*^2^ = 0.15) with 80% power and alpha level of 0.05. |

Continued on next page

| Quantitative variables | 11 | Explain how quantitative variables were handled in the analyses. If applicable, describe which groupings were chosen and why | 7 | Statistical analyses were conducted using SPSS statistical software version 29. |
| --- | --- | --- | --- | --- |
| Statistical methods | 12 | (*a*) Describe all statistical methods, including those used to control for confounding | 7 | Descriptive statistics were generated for all variables. A multiple linear regression was conducted to determine predictors of walking sport enjoyment. The regression model incorporated physical activity motivations, socio-demographic characteristics (e.g., age, gender), and health information (co-morbidities, BMI, perceived health). Assumptions for linear regression were not violated (i.e., independence of observations, linearity and homoscedasticity, normality, multicollinearity, and undue influence). |
|  |  | (*b*) Describe any methods used to examine subgroups and interactions |  | N/A |
|  |  | (*c*) Explain how missing data were addressed | 5 | A total of 325 individuals commenced the survey, of whom 31 did not provide data in relation to walking sport participation. This resulted in a final sample of 294 walking sport participants. |
|  |  | (*d*) *Cohort study*—If applicable, explain how loss to follow-up was addressed  *Case-control study*—If applicable, explain how matching of cases and controls was addressed  *Cross-sectional study*—If applicable, describe analytical methods taking account of sampling strategy |  | N/A |
|  |  | (*e*) Describe any sensitivity analyses |  | N/A |
| Results | | | | |
| Participants | 13* | (a) Report numbers of individuals at each stage of study—eg numbers potentially eligible, examined for eligibility, confirmed eligible, included in the study, completing follow-up, and analysed | 8-9 | Participant numbers are presented in Table 1. |
|  |  | (b) Give reasons for non-participation at each stage |  |  |
|  |  | (c) Consider use of a flow diagram |  |  |
| Descriptive data | 14* | (a) Give characteristics of study participants (eg demographic, clinical, social) and information on exposures and potential confounders | 8-9 | Participant characteristics (e.g., age, gender, marital status, BMI) are presented in Table 1 |
|  |  | (b) Indicate number of participants with missing data for each variable of interest | 8-9 | Table 1. |
|  |  | (c) *Cohort study*—Summarise follow-up time (eg, average and total amount) |  |  |
| Outcome data | 15* | *Cohort study*—Report numbers of outcome events or summary measures over time |  |  |
|  |  | *Case-control study—*Report numbers in each exposure category, or summary measures of exposure |  |  |
|  |  | *Cross-sectional study—*Report numbers of outcome events or summary measures | 5-7 | Outcome measures are described. |
| Main results | 16 | (*a*) Give unadjusted estimates and, if applicable, confounder-adjusted estimates and their precision (eg, 95% confidence interval). Make clear which confounders were adjusted for and why they were included | 12 | Presented in Table 4. |
|  |  | (*b*) Report category boundaries when continuous variables were categorized |  | N/A |
|  |  | (*c*) If relevant, consider translating estimates of relative risk into absolute risk for a meaningful time period |  | N/A |

Continued on next page

| Other analyses | 17 | Report other analyses done—eg analyses of subgroups and interactions, and sensitivity analyses |  |  |
| --- | --- | --- | --- | --- |
| Discussion | | | | |
| Key results | 18 | Summarise key results with reference to study objectives | 13-17 | Discussion |
| Limitations | 19 | Discuss limitations of the study, taking into account sources of potential bias or imprecision. Discuss both direction and magnitude of any potential bias | 17 | There are several limitations that must be acknowledged. We exclusively recruited current or recent walking sport participants, and this precluded comparisons with non-walking sport participants. This study also incorporated a very physically active (and motivated) sample, and this may limit the generalisability of the findings. |
| Interpretation | 20 | Give a cautious overall interpretation of results considering objectives, limitations, multiplicity of analyses, results from similar studies, and other relevant evidence | 17 | Overall interpretation is presented in Conclusion. |
| Generalisability | 21 | Discuss the generalisability (external validity) of the study results | 17 | Limitations in relation to generalisability are acknowledged. |
| Other information | |  | | |
| Funding | 22 | Give the source of funding and the role of the funders for the present study and, if applicable, for the original study on which the present article is based | 18 | Source of Funding: None |

*Give information separately for cases and controls in case-control studies and, if applicable, for exposed and unexposed groups in cohort and cross-sectional studies.

**Note:** An Explanation and Elaboration article discusses each checklist item and gives methodological background and published examples of transparent reporting. The STROBE checklist is best used in conjunction with this article (freely available on the Web sites of PLoS Medicine at http://www.plosmedicine.org/, Annals of Internal Medicine at http://www.annals.org/, and Epidemiology at http://www.epidem.com/). Information on the STROBE Initiative is available at www.strobe-statement.org.
